# Collective Motion of Spherical Bacteria

**DOI:** 10.1371/journal.pone.0083760

**Published:** 2013-12-20

**Authors:** Amit Rabani, Gil Ariel, Avraham Be'er

**Affiliations:** 1 Zuckerberg Institute for Water Research, The Jacob Blaustein Institutes for Desert Research, Ben-Gurion University of the Negev, Sede Boqer Campus, Midreshet Ben-Gurion, Israel; 2 Department of Mathematics, Bar-Ilan University, Ramat Gan, Israel; Semmelweis University, Hungary

## Abstract

A large variety of motile bacterial species exhibit collective motions while inhabiting liquids or colonizing surfaces. These collective motions are often characterized by coherent dynamic clusters, where hundreds of cells move in correlated whirls and jets. Previously, all species that were known to form such motion had a rod-shaped structure, which enhances the order through steric and hydrodynamic interactions. Here we show that the spherical motile bacteria *Serratia marcescens* exhibit robust collective dynamics and correlated coherent motion while grown in suspensions. As cells migrate to the upper surface of a drop, they form a monolayer, and move collectively in whirls and jets. At all concentrations, the distribution of the bacterial speed was approximately Rayleigh with an average that depends on concentration in a non-monotonic way. Other dynamical parameters such as vorticity and correlation functions are also analyzed and compared to rod-shaped bacteria from the same strain. Our results demonstrate that self-propelled spherical objects do form complex ordered collective motion. This opens a door for a new perspective on the role of cell aspect ratio and alignment of cells with regards to collective motion in nature.

## Introduction

Motile bacteria exhibit a large variety of motility mechanisms, among which some require cooperation between thousands of cells. Recent interest has been particularly given to such collective types of motilities, which were observed either in suspensions [1]–[8], or during migration and colonization of soft and hard surfaces [9]–[35]. Past work shows a growing body of research on many bacterial species including, *Serratia marcescens* [13]–[15], *Bacillus subtilis* [14]–[18], *Escherichia coli* [19]–[21], *Salmonella typhimurium* [21]–[22], *Paenibacillus dendritiformis* [23]–[26], *Paenibacillus vortex* [27], *Proteus mirabilis* [28], *Myxococcus xanthus* [29]–[32] and *Pseudomonas aeruginosa* [34]–[35]; all utilize different methods of motion such as swarming, twitching (social gliding), and ZBN swimming (collective motion at extreme cell densities). Common to all is the elongated, rod-like shape of the cells.

Cell elongation is one of the prominent qualities of bacterial swarming [9]–[11] - the most rapid surface migration mechanism. Swarming generally involves an organized, hyperflagellated-based, cell motion and a collective secretion of surfactants that decrease surface tension, thus enabling fast expansion. This motion has been studied extensively for different species [9]–[28] where some colonies can cover an entire Petri dish (8.8 cm in diameter) within a few hours. During such fast expansion bacteria move in whirls and jets in which hundreds of rod-shaped cells move in semi-circular patterns. Theoretically, there is no consensus whether cell elongation is necessary for collective motion. Recent work on "self propelled rods" suggests that the aligning interaction between the elongated *rod-like* shaped bacterial cells consists of hydrodynamic [1]–[8], [17], [23], [24], [25], [36]–[38] and steric interactions [17], [39]–[43]. Both types of interaction depend on the aspect ratio of swarmers (or collectively swimming bacteria), which is not present in spherical cells. On the other hand, some models [5], [38], [44] predict that collective motion of spherical bacteria is feasible. Since swarming was never observed in sphere-like bacteria, collective motion of such cells was questioned.

Collective bacterial motion is not limited to surfaces and may occur in liquids, but the physical and biological mechanisms involved in such processes may differ. Dombrowski et al. [2] found that highly dense suspensions (20–100 times more crowded than spontaneous overnight cultures) of *B. subtilis*, a rod-shaped bacterial species, swimming in sessile drops, show collective motion and flow patterns not found in control experiments with inert microspheres. Cisneros et al. [6] studied bacterial motion (*B. subtilis*) as a function of cell density and showed a transition to high speeds and directional order at high concentrations. Sokolov et al. [4] presented experimental studies of collective bacterial swimming (*B. subtilis*) in thin fluid films (1 µm), where the dynamics was essentially two-dimensional and the concentration could be adjusted continuously. At concentrations near the maximum allowed by steric repulsion, swimming bacteria formed a dynamic state exhibiting extended spatiotemporal coherence.

The reported collective bacterial motion in liquids has been limited for *B. subtilis*, a rod-shaped bacterial species. Since experiments were preformed at extremely high concentrations, geometrical restrictions arising from the aspect-ratio of the cells played a significant part in the explanations offered for the collective motion. Supporting this approach, two recent theoretical studies argued that spherical cells will not exhibit collective motion if only steric interactions will be considered. Wensink et al. [42] showed that for spherical cells, motion can be either free-swimming at low densities, or a jammed state at medium to high densities. Peruani et al. [43] showed that the transition to dynamic clustering (group behavior) depends on both the density and the cell aspect ratio, with no clustering for spherical cells. In particular, there is no transition to directional order with spherical bacteria. A key question to be discussed later is what exactly is a spherical bacterium – just the cell body or the entire bacterium with flagella combined.

In this work, we show that sphere-like bacteria do exhibit robust collective dynamics and correlated coherent motion while grown in suspensions. The 1 µm in diameter spherical cells of an overnight suspension of *S. marcescens*, swimming in a sessile drop, migrate to the upper surface of the drop, form a monolayer, and swirl in whirls and jets. The motion was found to strongly depend on cell density having a non-monotonic relationship between the mean cell swimming speed and cell concentration. Our results are essentially different from those obtained by others such as Dombrowski et al. [2] in several aspects: (i) The shape of the cells, which is spherical, (ii) Motion is restricted to a two-dimensional monolayer, (iii) A natural formation of cell density (no sample manipulation such as concentration by 20 fold), (iv) Gravity and buoyancy do not play a role. Our results demonstrate that, in contrast to previous hypotheses, the rod-like bacterial shape (aspect ratio >5) is not required for collective bacterial motion in liquids.

## Materials and Methods

### Strain and growth media

The wild type (WT) *Serratia marcescens* 274 is a Gram-negative motile bacterial species [13]. During the exponential growth phase (in broth), and at low densities (<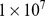), the cells have a rod-like shape (1×3 µm) and they swim in the volume at speeds of the order of 15 µm/s (run-and-tumble). In overnight cultures, at the stationary phase (18 h), the cells reach densities of 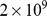
and shrink to the shape of spheres (possibly due to starvation [45]). The bacteria were maintained at –80°C in Luria Broth (LB) (Sigma, St. Louis, MO) with 25% [wt/vol] glycerol. LB broth was inoculated with the frozen stock and grown for 18 h at 30 °C while shaking (3 ml LB in a 15 ml plastic tube; at 200 RPM). It was subsequently grown to an OD_650_ of 2.0, corresponding to approximately 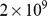 bacteria/ml, calibrated by counting colonies on agar after appropriate dilution. The counting-agar-plates were filled with 2% Difco agar from Becton Dickinson, supplemented with LB; culture dilution yielded 200±20 (after 5 independent repetitions) tiny colonies on each plate. OD measurements were done by using a basic spectrophotometer (Novaspec III), and standard 1 ml cuvettes; the suspensions were first diluted 1∶10 in LB to obtain a more reliable result. Experiments with rod-shaped cells, of aspect ratio of ∼3, were performed by diluting the 18 h old sphere-cells culture (100 µl into 3 ml of fresh LB), and growing them for 2 more hours in shaking.

Other *S. marcescens* strains used in this study are RH1041, which is a serrawettin mutant of *S. marcescens* 274 (no surfactant production (SMu 13a)), RH1037 which is an immotile mutant of *S. marcescens* 274 (flagella minus (Hag::Cm)), and *S. marcescens* strain *A* [15] which is a WT that produces much more surfactant than the WT 274 strain. To obtain supernatants, cultures were centrifuged at 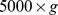 for 3 min (high speed limits the amount of remaining cells), then the liquid was harvested. For mixing of centrifuged cells with supernatant, the cells were centrifuged at 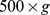 for 1 min (low speed prevents damage to the centrifuged cells), their supernatant was removed, and the desired supernatant was added by careful pipette mixing.

### Microscopic measurements

An optical microscope (Zeiss Axio Imager Z2) equipped with a LD 60X Phase Contrast objective lens was used to follow the microscopic motion. The microscope was placed in a temperature and humidity controlled environment. A digital camera (GX 1050, Allied Vision Technologies) captured the microscopic motion at a rate of 100 frames per second and a spatial resolution of 1024×1024 pixels. Movies were taken for 20 min periods, streamed directly to the hard drive, resulting in 120,000 images in a sequence.

The fraction of area occupied by bacteria, *ρ*, was calculated by measuring the number of black pixels covering the frame, then divided by the entire number of pixels (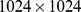). See Fig. S1 for additional details.

### Flow analysis

Recorded movies were converted to a sequence of single-frame images. Following standard pre-processing for noise reduction, the optical flow between each two consecutive frames was obtained using the Horn-Schunk method [46]. Vector fields were reduced to a 64×64 grid by simple averaging, generating an approximated velocity field 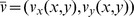. In order to characterize the observed flow patterns, several other dynamical variables were calculated

- The vorticity field 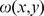
defined as the *z*-component of the curl of 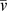.
- Spatial correlation functions

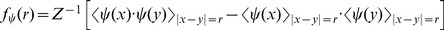



where *Z* is a normalization constant such that 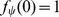, 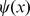 is a given field and 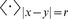 denotes averaging over all pairs of grid points *x* and *y* separated by *r* and over all frames with concentration in a given range.

- Temporal correlation functions

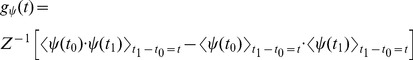



where *Z* is a normalization constant such that 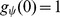, 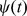 is a given field and 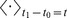 denotes averaging over all grid points and times 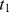 and 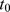 such that 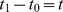.

In particular, we are interested in correlations in directions 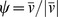 denoted 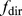 and correlations in vorticity 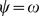 denoted 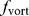. Similar notations are used for temporal correlation functions, 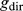 and 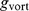, respectively. Averages at fixed concentrations are taken over all spatial positions and all frames within the 5 seconds corresponding to the specific concentration.

## Results

### Observation of collective dynamics

**Figure 1:**
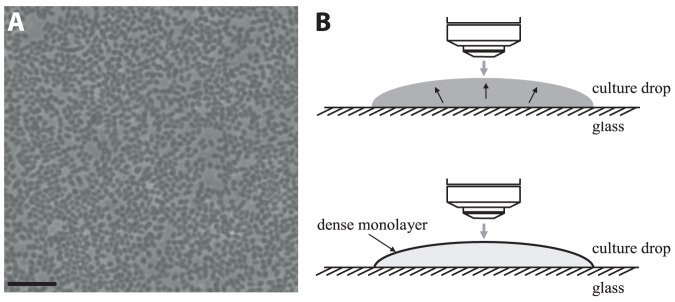
Observation of the collective dynamics of WT *S. marcescens* 274 bacteria, swirling in a drop. (**A**) A snapshot of an overnight (18 h) culture-drop placed on a glass slide; image taken by an upright phase contrast microscopy. All cells are spherical and are located on the upper surface of the drop. Bacterial density (fraction of area occupied by bacteria) *ρ* = 0.67. Scale bar equals 10 µm. (**B**) A schematic side view, illustrating how cells swim from a homogeneously dense volume to the upper surface of the drop, forming a dense monolayer and an extremely sparse bulk.

A 5-µl drop of an overnight (18 h) WT *S. marcescens* 274 culture was placed on a glass slide and observed in upright light microscopy (phase contrast). Bacterial density (per volume) for such a culture was 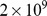 cells/ml. All cells were spherical with an aspect ratio smaller than 1.1 (some were slightly oval because of pre-splitting due to reproduction) (Fig. 1A), and were constantly swimming from the bulk to the upper surface of the drop. The cells remained at the upper surface, thus continuously increasing the surface cell density (Fig. 1B). Increment of surface density from minimal to maximal lasted approximately 20 min. On the surface, cells formed a monolayer and were swirling in dynamic whirls and jets (Movie S1). The motion was found to be independent of either the material from which the surface was made or the size of the drop; it was also independent of the droplet’s surface shape, either concave, nearly flat or convex. For control, some drops were placed on the glass, then tilted upside-down and viewed by an inverted microscope to see the effects of gravity and buoyancy. No change was observed. For quantitative measurements the drop was constrained by a super-hydrophobic ring printed on the glass (polytetrafluoroethylene (PTFE) printed glass slides (63429-04) Electron Microscopy Sciences, Hatfield, PA) in order to prevent wetting and spreading, which may affect the dynamics of the bacteria or cause drifting. To prevent both evaporation and the blowing of air on the sample the drop was enclosed in a small chamber, the top and bottom of which comprised of thin glass coverslips, while the surrounding wall was a metallic ring attached to the glass with vacuum grease (Fig. S2).

### Scaling of the bacterial dynamics

**Figure 2:**
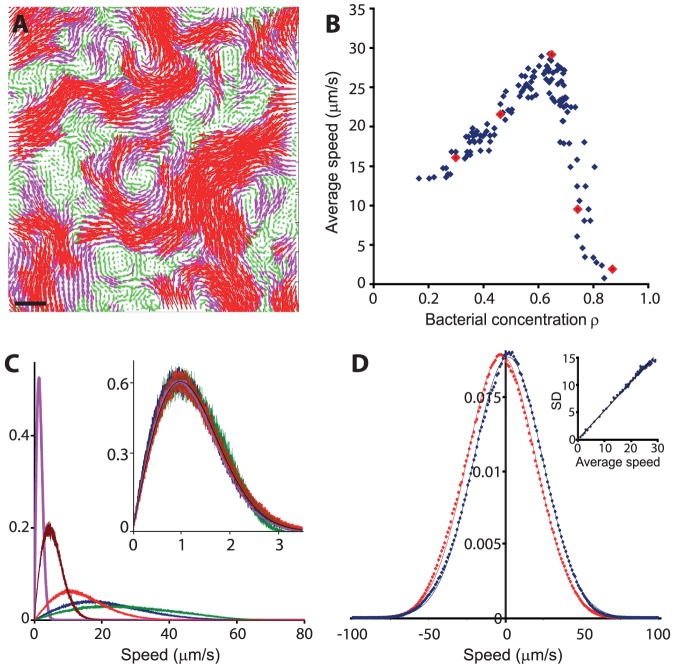
Velocity field analysis. (**A**) The velocity field at *ρ* = 0.67. Length of arrows represents a local speed, also manifested by color: <20 µm/s green, >20 and <40 µm/s pink, and >40 µm/s red. Scale bar equals 10 µm. (**B**) The average bacterial speed as a function of bacterial concentration *ρ*. The speed reaches a maximum at *ρ* = 0.64. Red large dots represent 5 different concentrations discussed in (C). (**C**) Probability density histogram for 5 different bacterial concentrations *ρ* = 0.28 red, 0.46 blue, 0.67 green, 0.74 brown and 0.87 pink; the *y*-axis is normalized so that the area below each curve equals 1. Inset - All speed distributions are well approximated by a Rayleigh distribution, the black solid line. (**D**) The probability density of velocity projections on the principal axes are approximately independent normal distributions with zero mean; *x*-axis blue, *y*-axis red; dots represent experimental data and solid line is the Normal distribution. Inset - the standard deviation of the speed grows linearly with the average speed with a slope of approximately 0.52 (axes are inµm/s).

To quantify the dynamics of the sphere-like collectively swimming bacteria, we calculated a velocity field for each two consecutive frames. A typical raw movie lasts ∼20 min; the data was taken from 5 similar experiments, ending up with about 600,000 analyzed frames. Each movie was divided into 5-second segments, in which the bacterial density was approximately constant. Figure 2A depicts a typical velocity field, showing the velocity field at a typical bacterial concentration *ρ* = 0.67. See also Movie S2. Figure 2B shows the average bacterial speed as a function of the bacterial concentration in each 5-second segment; At low densities, the mean speed is an increasing function, reaching a maximum at *ρ* = 0.64. At higher densities, the mean speed decreases sharply to a jammed high-concentrated phase. Figure 2C shows the distribution of speeds at 5 concentrations (*ρ* = 0.28, 0.46, 0.67, 0.74 and 0.87), also denoted in Fig. 2B as red dots. Interestingly, all speed distributions are well approximated by a Rayleigh distribution (see inset in Fig. 2C) with probability density 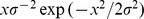, where 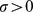 is a parameter indicating the point in which the maximum of the density is obtained (the mode). The mean of the Rayleigh distribution is 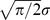 and the standard-deviation is 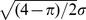. Thus all Rayleigh distribution collapse to a master curve with 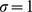 upon rescaling by 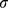. The Rayleigh distribution is a consequence of the fact that projections of velocities on the principal axes are approximately independent normal distributions with zero mean and variance 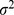 (Fig. 2D). We demonstrate this scaling by showing that the standard deviation of the speed distribution grows linearly with the average speed with a slope of approximately 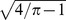 (∼0.52).

**Figure 3:**
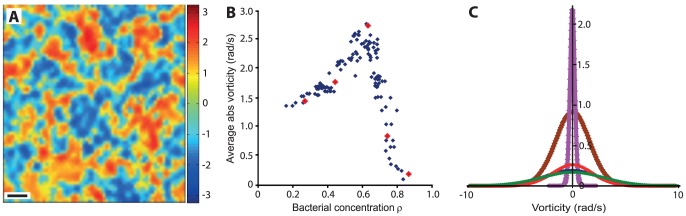
Vorticity field analysis. (**A**) The vorticity field at *ρ* = 0.67. Colors indicate the *z*-component (the magnitude) of the curl of the velocity field - the "strength" of the vorticity. Values on the bar are in rad/s. Negative values (blue) means clock-wise motion and positive values (red) means counter clock-wise motion. Scale bar equals 10 µm. (**B**) The average absolute value of vorticity as a function of bacterial concentration *ρ*. The red large dots represent the 5 different concentrations discussed in (C). (**C**) Probability density histogram for 5 different bacterial concentrations *ρ* = 0.28 red, 0.46 blue, 0.67 green, 0.74 brown and 0.87 pink; the *y*-axis is normalized so that the area below each curve equals 1. All speed distributions are well approximated by a Normal distribution.

**Figure 4:**
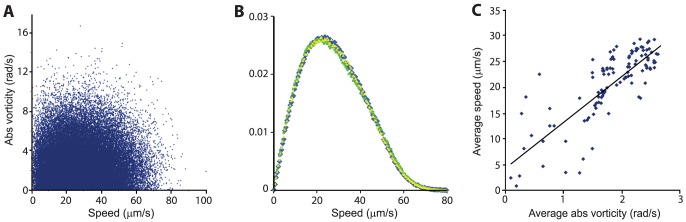
Correlation between mean speed and vorticity. (**A**) At a fixed concentration (here *ρ* = 0.67), the speed and the vorticity are uncorrelated with R^2^ = 0.02. (**B**) Probability density of speeds at three different vorticity ranges (terciles), represented in yellow, green and blue, is the same (*ρ* = 0.67). (**C**) The average speed as a function of the average absolute value of the vorticity. The two are highly correlated (R^2^ = 0.65).

The vorticity field 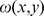
shows a similar scaling with *ρ*. Figure 3A shows the vorticity field at the same typical concentration *ρ* = 0.67. See also Movie S3. Figure 3B shows the average absolute value of vorticity as a function of the bacterial concentration (note the similarity to Fig. 2B). Figure 3C shows the distribution of the vorticity at 5 concentrations (*ρ* = 0.28, 0.46, 0.67, 0.74 and 0.87) denoted in Fig. 3B as red dots. It is approximately normal. At a fixed concentration, speed and vorticity are uncorrelated (0.02 at *ρ* = 0.67, see Fig. 4A). Figure 4B shows that the distribution of speeds at three different vorticity ranges (terciles) is the same, implying that cells that move in jets, and cells that move in whirls have same speed distribution. However, the standard deviation in the normal vorticity distribution (and therefore also the average absolute vorticity) depends on concentration (and therefore on σ and the mean speed). Figure 4C shows the average speed as a function of the average absolute value of the vorticity. The two are highly correlated (0.65 at *ρ* = 0.67) with a slope of 8.8.

**Figure 5:**
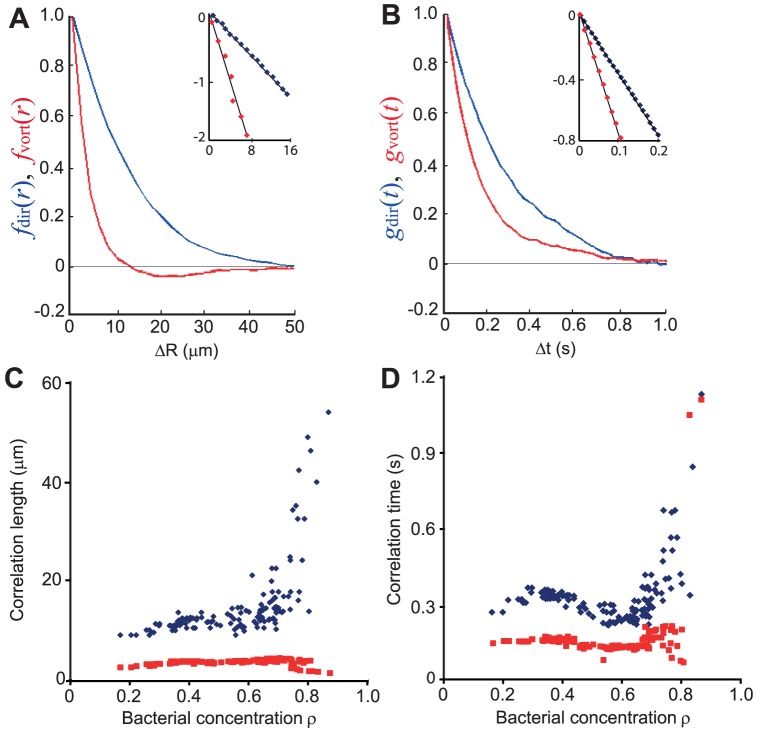
The spatial and temporal correlation functions. (**A**) An example for *ρ* = 0.67; The spatial correlation function of the velocity (blue) and vorticity (red) functions show exponential decays (see straight lines in the semi-log inset). (**B**) same for the temporal correlation functions. (**C**) The correlation length (blue for velocity and red for vorticity) as a function of bacterial concentration. (**D**) The correlation time (blue for velocity and red for vorticity) as a function of bacterial concentration.

Both the spatial and temporal correlation functions were found to decay exponentially, thus defining a characteristic decay length 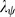 and time 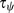. Figures 5A-B depict the spatial and temporal correlation functions, respectively, at *ρ* = 0.67. Figures 5C-D show the correlation lengths and times as a function of bacterial density. While the average speed changes dramatically, both 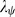 and 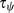 remain fairly constant; *λ*_dir_ and *τ*_dir_ diverge at very large bacterial concentrations where the speed is small and the dynamics is jammed. The vorticity correlation function 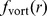 becomes negative around the characteristic size of the whirls, which is approximately 20 µm. The vorticity correlation time is about 0.15 s, indicating very short-lived vortices.

The scaling described above suggests that the bacterial dynamics is the same at all concentrations up to a single multiplicative constant 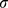. The scaling extends from the lowest measured concentration *ρ* = 0.18 up to the jammed phase observed for *ρ* = 0.87.

### Comparing spherical and rod-like bacteria

**Figure 6:**
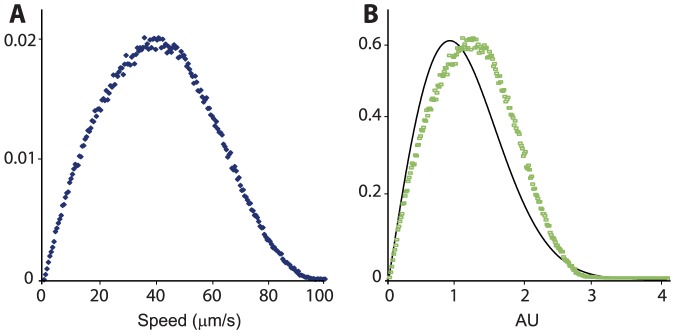
Rod-shaped cells. (**A**) Probability density of speeds shows a much faster speed compared with those obtained for spherical cells (Fig. 2C). The *y*-axis is normalized so that the area below the curve equals 1. (**B**) Same data as in (A) now normalized vs. a Rayleigh distribution, the black solid line.

So far we have shown that sphere-like bacteria exhibit strong collective dynamics. But how different is this motion if compared to rod-shaped cells of the same species? We have thus repeated the entire experiment with similar, but longer cells (aspect ratio of ∼3; see Material and Methods). For the long cells the mean speed and vorticity were significantly larger (+35%) (Fig. 6A), while correlation lengths and times were the same. A striking result was that the long cells did not show the Rayleigh speed distribution observed for the spherical cells (Fig. 6B). Instead, the long cells showed a narrower distribution with a faster decaying tail (analyzing the dynamics of *B. subtilis*, a rod-shaped bacterial species with an aspect ratio of approximately 4.8, Wensink et al. [42] found that projections of the bacterial velocity of the principal axes is roughly Gaussian, pertaining to a Rayleigh speed distribution). These differences support the idea that the long cells do benefit from large hydrodynamic and/or steric interactions that lead to a much faster motion, and to a more concentrated speed distribution. Also it shows that the bacterial shape does not dictate the correlation time and length (*λ* and *τ*) as suggested for *B. subtilis* by Sokolov and Aranson [8].

### Role of chemical signaling and surfactants

Collective swirling of the cells may be triggered or enabled by secretion of surfactants or other compounds unique to *S. marcescens*, thus modifying of the physical properties of the drop. For example, it was shown [15] that the mobility of the upper surface of surfactant producing bacterial swarms is significantly different from those of non-surfactant producers. In order to test the origin of the motion in this experiment, we have used different strains of *S. marcescens*: (i) a motile strain that does not produce surfactants (RH1041), (ii) a motile strain (strain *A*) that produces larger amounts of surfactant (compared to WT 274) and (iii) an immotile strain that produces surfactant (RH1037). The results showed that immotile bacteria did not migrate to the surface and did not show collective motion. Furthermore, they did not show individual swimming, but Brownian motion was evident. The two motile strains, one that does not produce surfactants and the other that produces large amounts of surfactants, both showed the same collective motion as the WT 274. We thus suggest that collective motion is independent of surfactant production and that, instead, it stems from the intrinsic motility properties of each cell.

Next, we moved motile cells into supernatant of immotile cells, and immotile cells into supernatant of motile cells. The motile cells swirled in the immotile supernatant and the immotile cells did not move in the motile supernatant (beside Brownian motion). When we moved the motile cells into fresh LB broth they did not show the swirling pattern; some cells were swimming individually. We thus suggest that the collective swirling behavior is a result of both self propulsion, and the presence of some molecule that is not a motility-associated material.

## Discussion

Alignment of individuals within a group was found to play a significant role in the dynamics of many species and artificial agents [47]. Naturally, the geometry of objects plays an important role in the effective aligning interaction. For example, steric interaction between inanimate rod-shaped particles (e.g., rice seeds) can lead to non-equilibrium and collective behavior [48]–[50]. However, no ordered phase was found in inanimate vibrating round particles, both in experiments and simulation [50].

The question of whether motile spherical bacteria can form collective motion is undecided. Recently, two theoretical models did not find any ordered phase in systems of self-propelled spherical particles [42]–[43] and concluded that the transition to collective behavior strongly depends on the aspect ratio of the particles. On the other hand, it may be argued that even spherical motile cells cannot be considered spherical because of the long flagella at their poles. Indeed, several models simplify the complex shape of bacteria as a sphere attached to a thin rod, or a force dipole representing the flagellar bundle [5], [51]. However, several *experimental* works have shown that the flagella do not behave like a rod attached to a sphere, and that flagella are easily deformed; hence, self-propelled spherical cells are effectively spherical and not rods. By using fluorescently labeled flagella, Turner et al. [52] visualized the flagellar filaments of planktonic bacteria and showed that the bundle does not behave as a rigid rod hooked to the cell body. Both Turner et al. [53] and Copeland et al. [20] have found that the bundles of flagella on swarmer cells periodically splay apart, leading to the reorientation of the cell. In addition, Copeland et al. [20] observed that flagella make transient contact with flagella on adjacent cells. More importantly, it was found that swarmer cells spend a considerable amount of their time in the community in a passive state responding to the motion of adjacent cells. We thus suggest that the description of a bacterial cell to be composed of a sphere attached to a rod, may not necessarily be relevant to our experiment, as flagella cannot be considered an extension of the spherical body that makes it cylindrical-like. Non-the-less, it may be responsible for creating small asymmetries which may assist in the creation of collective motion. We stress that such asymmetries cannot explain the quantitative flow analysis of collective motion observed in experiments since the speed distribution of elongated bacteria was found to be fundamentally different than that of spherical ones.

Our experimental results suggest that spherical bacteria may form a collective state after all. Collective motion strongly depends on the concentration (Fig. 2B and Fig. 3B). The speed distribution of the cells followed a Rayleigh distribution (stemming from the fact that projections of velocities on the principal axes are approximately independent normal distributions) at all bacterial concentrations (Fig. 2C). This observation is particularly important since models of granular material show that at high densities, the speed distribution of granular gases in which particles interact inelastically decays exponentially. Hence, a Gaussian speed distribution for spherical bacteria can serve a benchmark test for different swarming or collective swimming models (e.g., [44], [51]).

In addition, vorticity depends on concentration but is independent of speed (Figs. 4A-B). In particular, collective behavior was evident at even low concentrations, where the distance between cells is large, reducing the contribution of steric and short-range hydrodynamic interactions. On the other hand, *rod-shaped* bacteria moved much faster and showed a different speed distribution (Fig. 6), other than Rayleigh. These results suggest that there are fundamental differences between rods and spheres, possibly due to the added rotational symmetry of spheres. In addition, the relatively constant correlation lengths and times, which are also similar for spheres and rods (Figs. 5C-D), suggest that the main factors responsible for the appearance of collective motion may be the physical properties of the medium, such as viscosity, and the diffusion properties of signaling molecules, rather than the geometry of the cells.

Indeed, testing the role of chemical signaling, we found it is plausible that a non-motility-associated chemical exists in the culture from early stages. However, we cannot conclude whether such a chemical generates the collective behavior, or simply enables it, acting like a trigger. The results of this work open a door for a new perspective on the role of cell aspect ratio and alignment of cells with regards to collective motion in nature.

## Supporting Information

Figure S1 **Calculating the bacterial density** ***ρ*** **.** (**A**) The raw image. (**B-C**) An intensity histogram for each frame was plotted. Two maxima were always obtained indicating the grey level for the cells and for the background. Threshold was determined based on the minima. (**D-F**) The uncertainty in determining the threshold was ±1 grey level, which led to the maximal uncertainty in *ρ* of ±0.03.
(PDF)

Figure S2 **The sample holder.** (**A**) Side-view schematics of the bacterial monolayer at the surface of a drop. (**B**) The drop is enclosed in a small chamber, the top and bottom of which comprised thin glass coverslips, while the surrounding wall is a metallic ring attached to the glass with vacuum grease. (**C**) and (**D**) Side and top views of the setup, showing the hydrophobic ring (brown) stamped on the bottom piece of glass to prevent the drop from spreading.
(PDF)

Movie S1 **A raw (compressed), real time movie of wild type** ***S. marcescens*** **274 bacteria, swirling on the upper surface of an overnight culture drop.** Frame size equals 60 µm. Each cell is approximately 1 µm in diameter. Average (over time) bacterial concentration *ρ* = 0.67.
(AVI)

Movie S2 **The velocity field of WT** ***S. marcescens*** **274 bacteria, swirling on the upper surface of an overnight culture drop.** Bacterial concentration *ρ* = 0.67. Length of arrows represents a local speed, also manifested by color: <20 µm/s green, >20 and <40 µm/s pink, and >40 µm/s red. Frame size equals 100 µm. Length of movie in real time is 2 s.
(MPG)

Movie S3 **The vorticity field of WT** ***S. marcescens*** **274 bacteria, swirling on the upper surface of an overnight culture drop.** Bacterial concentration *ρ* = 0.67. Colors indicate the *z*-component (the magnitude) of the curl of the velocity field - the "strength" of the vorticity. Dark blue represents clock-wise motion (up to ∼3 rad/s) and dark red represents counter clock-wise motion (up to ∼3 rad/s). Frame size equals 100 µm. Length of movie in real time is 2 s.
(WMV)
