# Supplementary figures and images for: Collective Motion of Spherical Bacteria
Source: PLoS One. 2013 Dec 20;8(12):e83760. doi: 10.1371/journal.pone.0083760 (PMC3869797; doi:10.1371/journal.pone.0083760)

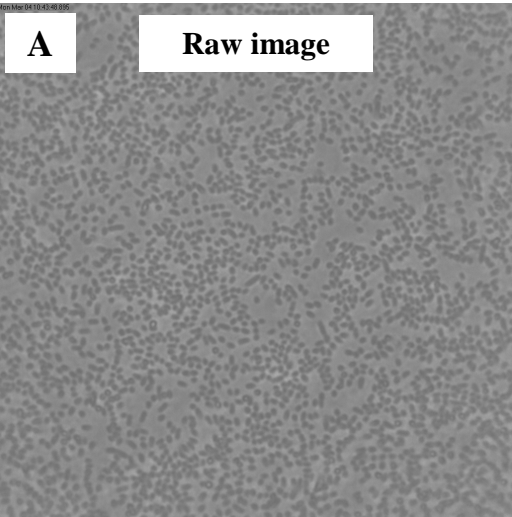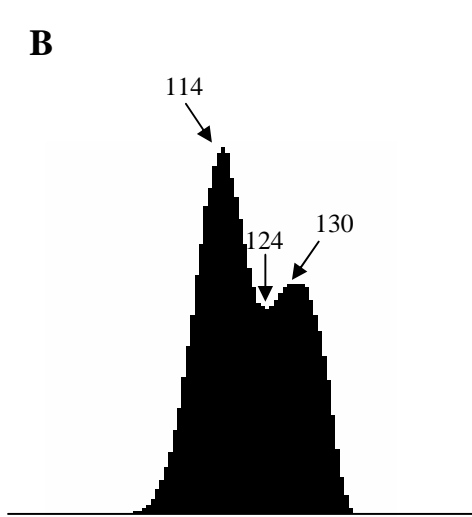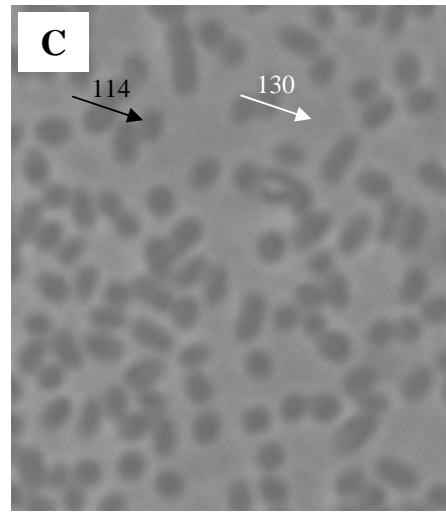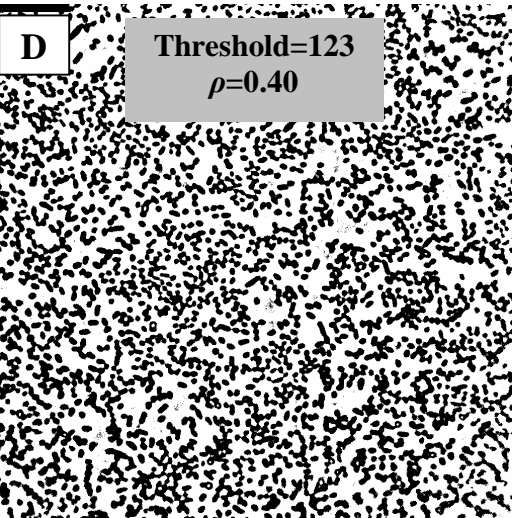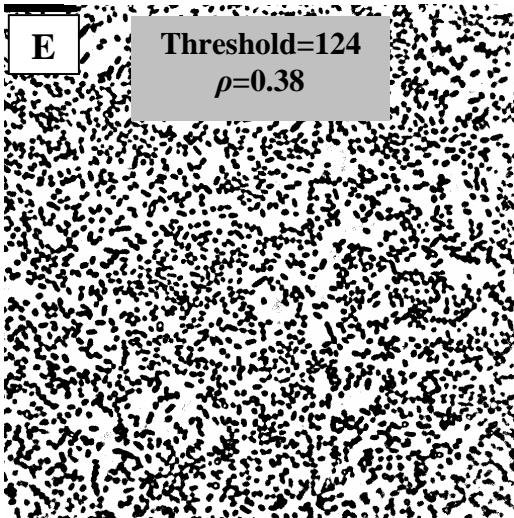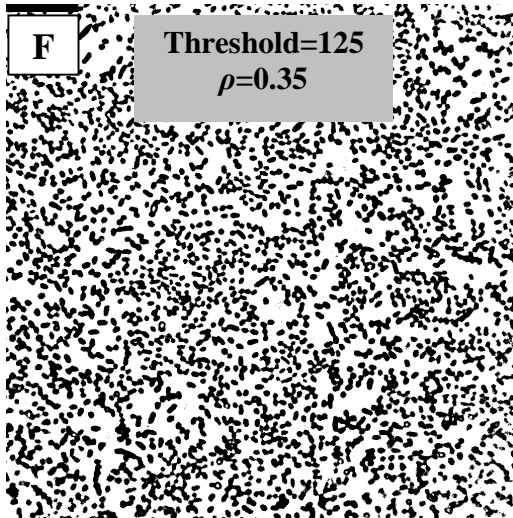

Supplement: Figure S1 — Calculating the bacterial density ρ . (A) The raw image. (B-C) An intensity histogram for each frame was plotted. Two maxima were always obtained indicating the grey level for the cells and for the background. Threshold was determined based on the minima. (D-F) The uncertainty in determining the threshold was ±1 grey level, which led to the maximal uncertainty in ρ of ±0.03. (PDF) [file pone.0083760.s001.pdf]

**A**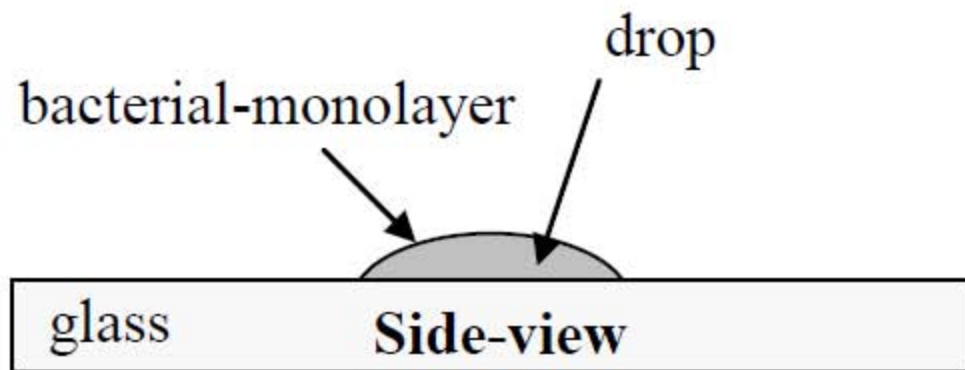**B**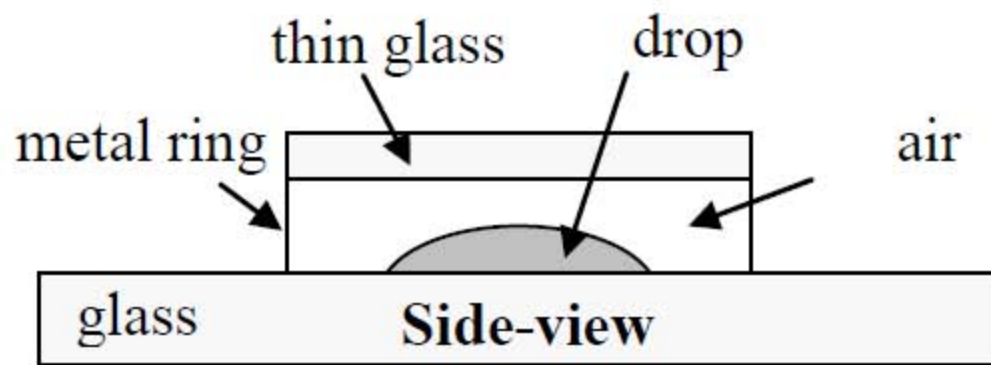**C**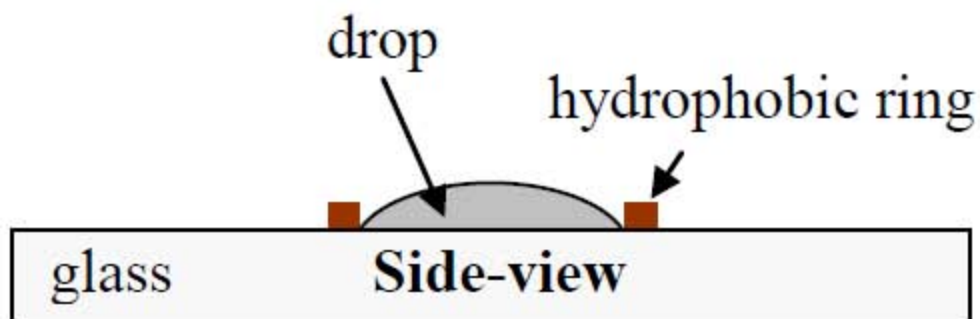**D**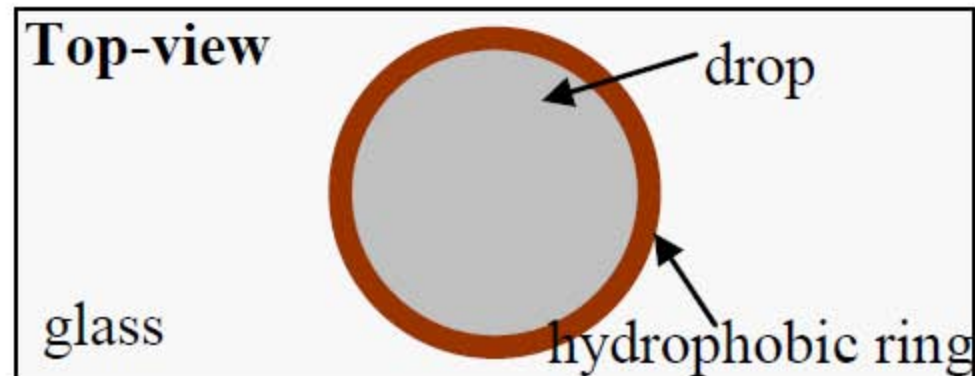

Supplement: Figure S2 — The sample holder. (A) Side-view schematics of the bacterial monolayer at the surface of a drop. (B) The drop is enclosed in a small chamber, the top and bottom of which comprised thin glass coverslips, while the surrounding wall is a metallic ring attached to the glass with vacuum grease. (C) and (D) Side and top views of the setup, showing the hydrophobic ring (brown) stamped on the bottom piece of glass to prevent the drop from spreading. (PDF) [file pone.0083760.s002.pdf]
